# Supplementary material for: Integration of landmark and saccade target signals in macaque frontal cortex visual responses
Source: Commun Biol. 2023 Sep 13;6:938. doi: 10.1038/s42003-023-05291-2 (PMC10499799; doi:10.1038/s42003-023-05291-2)
Supplement: Supplementary file 2 — Supplementary information [file 42003_2023_5291_MOESM2_ESM.pdf]

Supplementary Figures and Analyses

Integration of landmark and saccade target signals in macaque frontal cortex visual responses

Adrian Schütz[1,2]*, Vishal Bharmauria[3]*, Xiaogang Yan[3], Hongying Wang[3], Frank Bremmer[1,2]**, J. Douglas Crawford[3,4]**

* These authors contributed equally
**These authors jointly supervised this work

1. Department of Neurophysics, Phillips Universität Marburg, Marburg, Germany
2. Center for Mind, Brain, and Behavior – CMBB, Philipps-Universität Marburg, Marburg, Germany & Justus-Liebig-Universität Giessen, Giessen, Germany
3. York Centre for Vision Research and Vision: Science to Applications Program, York University, Toronto, Canada
4. Departments of Psychology, Biology, Kinesiology & Health Sciences, York University, Toronto, Canada

Corresponding author:
Dr. John Douglas Crawford
Departments of Psychology, Biology and Kinesiology & Health Sciences
York University, Toronto, Canada
Centre for Vision Research, Room 0009A LAS
4700 Keele Street, Toronto, Ontario, M3J 1P3
Email: jdc@yorku.ca
Phone: 416-736-2100 x 88621
Fax: 416-736-5857

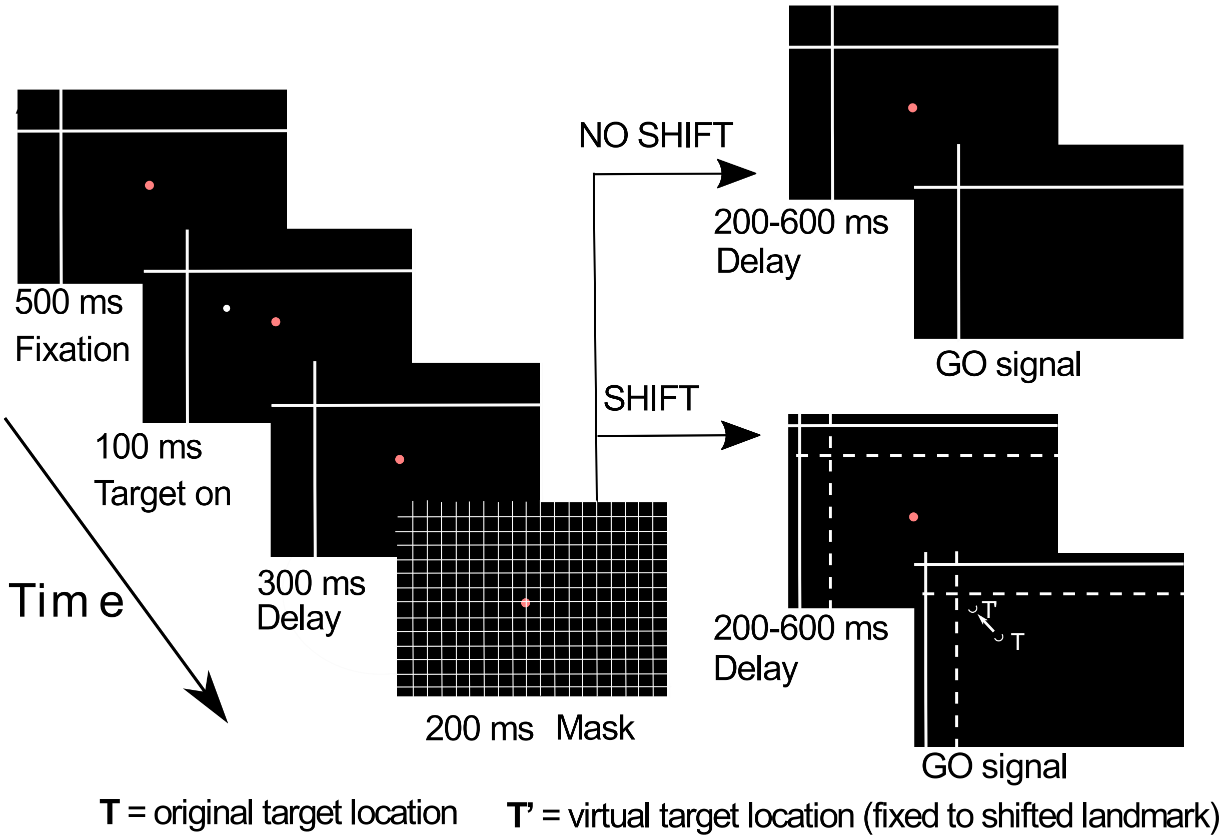

Supplementary Fig. 1: Complete memory-delay / landmark paradigm, showing the initial landmark-target combinations analyzed here, and cue-conflict and saccade ‘go’ signals that occurred later in the time course (not analyzed here). The monkey starts the trial by fixating a central red dot for 500 ms in the presence of two white intersecting lines (landmark). Then a white dot (target) is flashed (100 ms) in one of four possible locations relative to the landmark, followed by 300 ms delay (the first portion of this delay corresponds to the analysis period in this study) and a grid-like mask (200 ms). After mask offset the landmark shifted / not shifted in one of eight radial locations from the original location. Then after an additional delay period (200-600 ms) the fixation point was extinguished which served as a go signal for the animal to saccade to the remembered target location. Note that the latter event occurred well after the data described in the current study. The animal was rewarded for landing its gaze (G) within a circle of radius 8-12° around the original target, thereby being large enough to neither reward nor punish any implicit landmark influence on behavior.

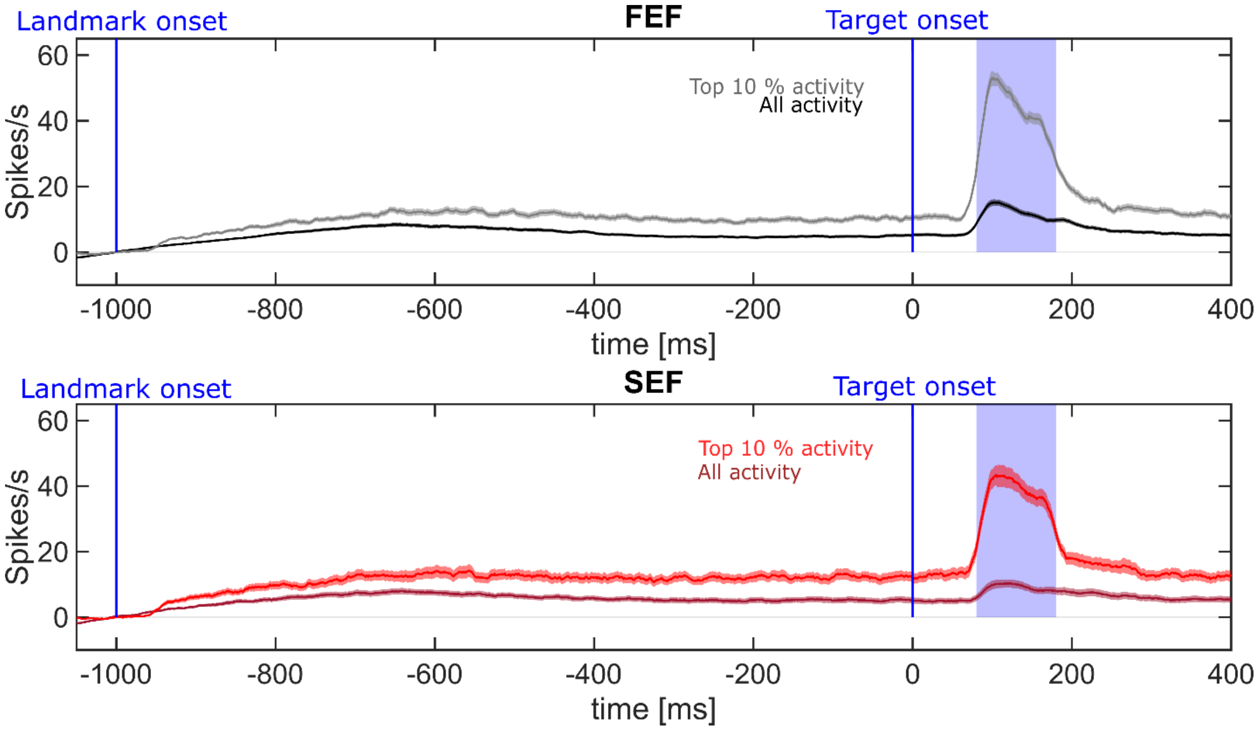

Supplementary Fig. 2: Population mean spike density plots for the FEF (top), and SEF (bottom) neurons aligned to the landmark onset (first vertical blue line). Note a sluggish response to landmark onset in both cases. The darker line corresponds to all the responses and lighter curves represent the top 10 % responses.

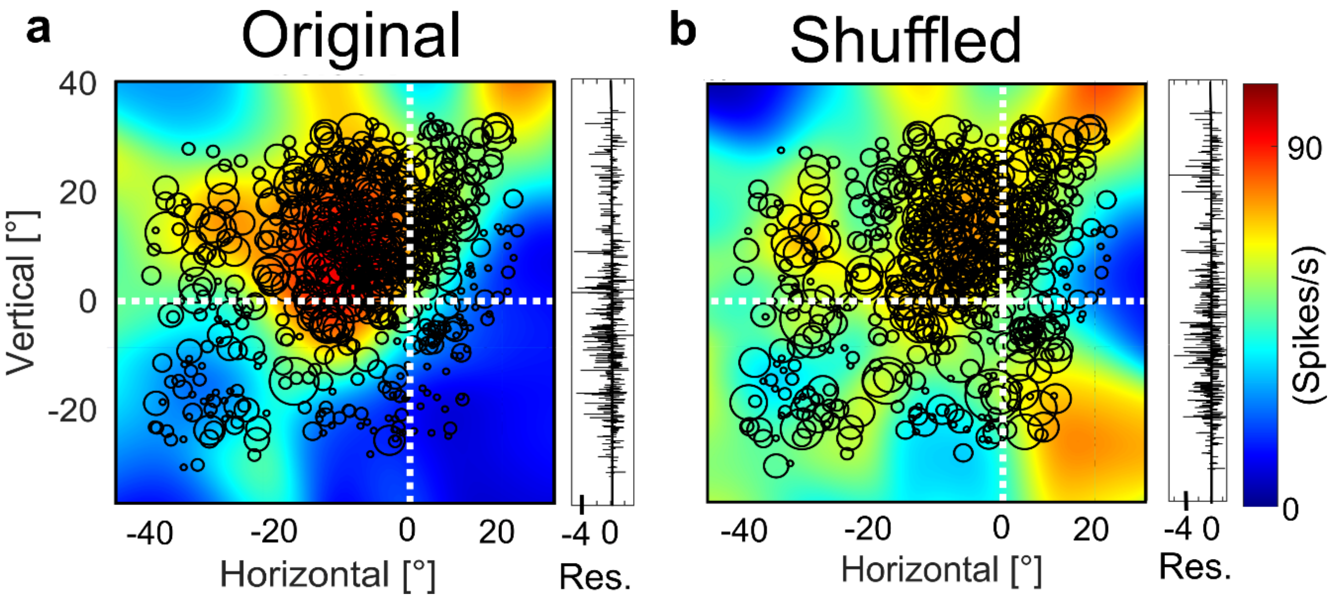

Supplementary Fig. 3: Comparison between a **a** real and the corresponding randomly **b** shuffled response field for an example FEF neuron (same neuron as in **Fig. 4c-e**). In the example shuffled response (1 of 100), the hot-spot is degraded, the distribution of ‘small and big responses’ is broader and more intermingled, and quantitatively the residuals show a larger spread between the data and the fit. Specific patterns vary for each shuffle.

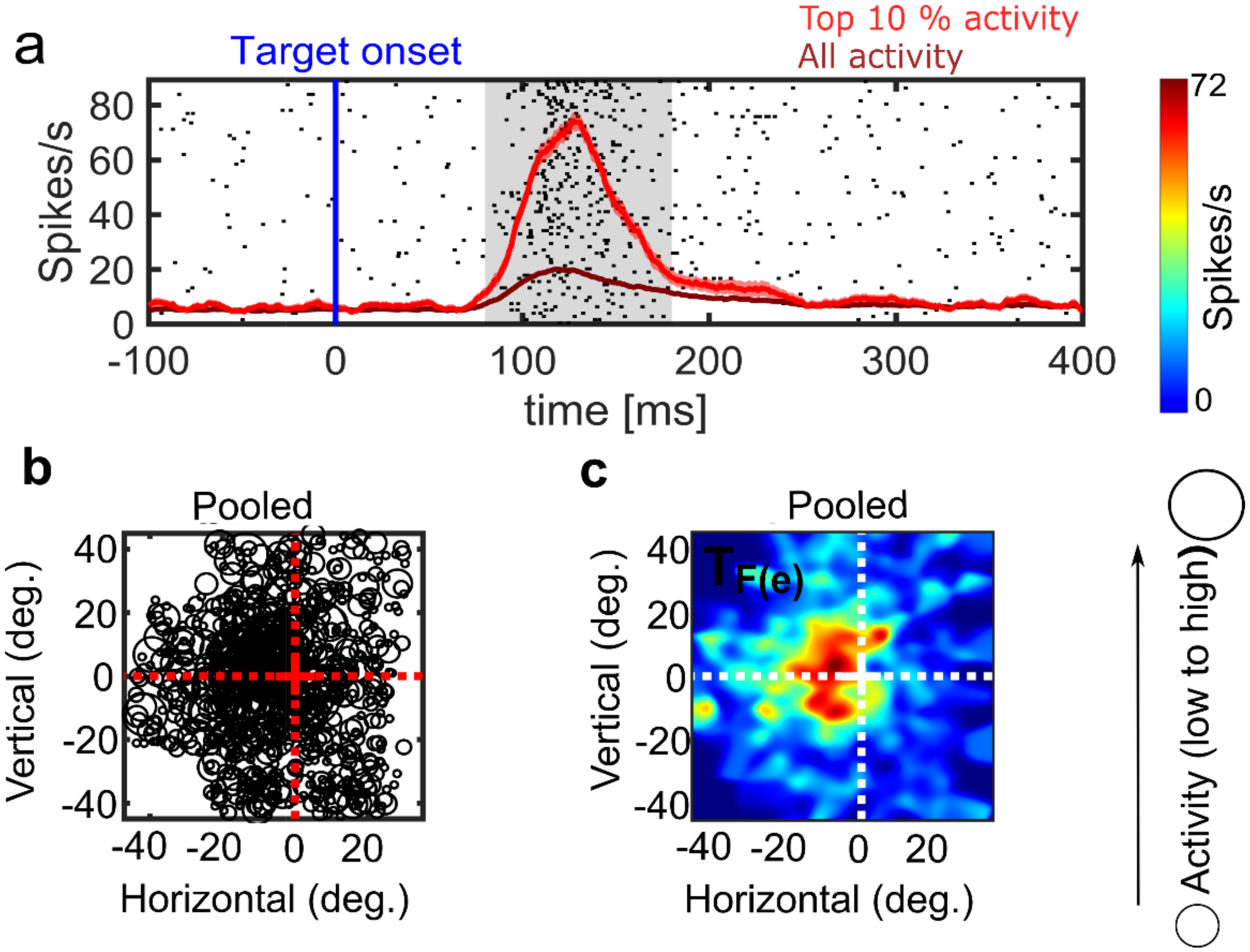

Supplementary Fig. 4: Typical example of a visual response analysis (for an SEF neuron). a Shows the raster of the neuronal activity. The vertical blue line indicates the target onset, and the grey shaded area represents the time window used for further analysis. The dark red line shows the activity density for all trials, the light red line shows the activity for the 10 % trials with the highest activity in the time window between 80 ms to 180 ms after target onset. The raster plot is shown for the top 10 % responses. **b** Fitting of neural responses for all target-landmark configurations. **c** Shows the non-parametric fit of the neuron's response field. The color scale on the right indicates activation ranging from low (blue) to high (red). The black circle shows the position of a trial in the feature space. The size of the circle represents the corresponding neuronal activation in this trial in the analyzed time window. The red / white cross denotes the center of the coordinate system (0,0).

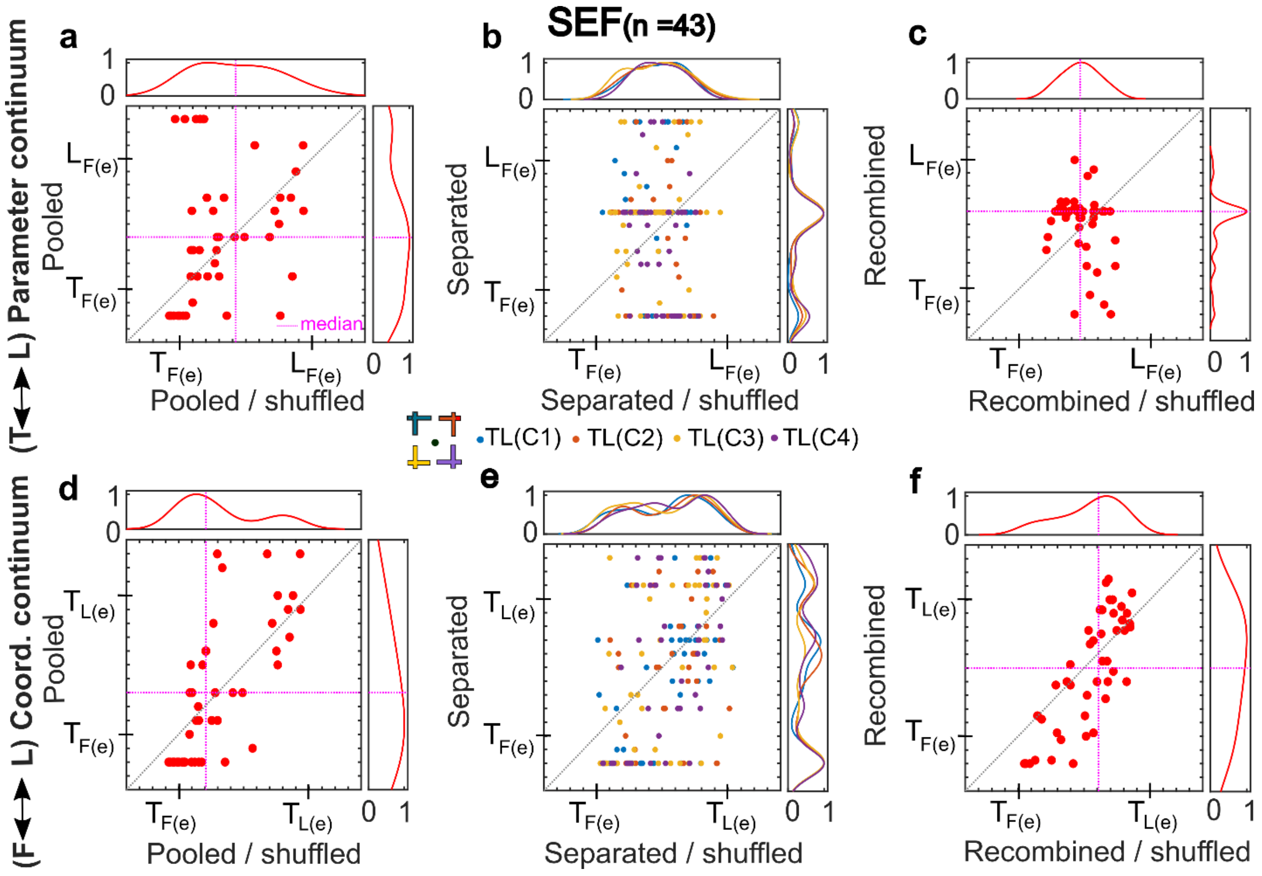

Supplementary Fig. 5: Continuum Analysis for SEF. a-f Complete Continuum analysis for all SEF neurons. In each panel, fits to experimental data are plotted as a function of the median best fit for their corresponding 100 shuffled control datasets, and their overall distributions are shown above / beside the plot. The magenta lines show the intersection of medians along x and y axes and the diagonal dotted lines show the line of equality between pooled and shuffled control fits. To be comprehensive, we included neurons with and without spatial tuning in this analysis. **a-c** Swarm plots of the distributions of best fits along the T-L Parameter continuum. **a** Fits for response fields where data were pooled across different target-landmark configurations. Again, like FEF, at the pooled level, SEF data did not display any significant deviation (Wilcoxon signed rank test, $p = 0.68$) between the original (mean = 0.35, median = 0.30, std = 0.51) and control (mean = 0.35, median = 0.34, std = 0.25) data at the population level, but their distributions were significantly different (10,000 bootstraps, 95 % confidence interval). Specifically, 13.6 % of the SEF neurons showed a significant landmark influence relative to their control data (95 % bootstrapped confidence interval). **b** Configuration-dependent analysis. Each color represents a specific target-landmark configuration. A similar trend as FEF was observed for SEF neurons, with 31.8 % of them being significant for at least one configuration, and different fractions of data being significant (*TLC1-4*: 9.1 % / 13.6 % / 11.4 % / 18.2 %) at

the level of individual target-landmark configurations. Each color represents a specific target-landmark configuration. **c** Recombined data (mean of 4 median fits / cell). **d-f:** Similar analysis (as c-e) but for the F-L Coordinate Continuum. **d** Fits for pooled configuration data. Again, like FEF, the influence in SEF was bidirectional, cancelling out at the population level (Wilcoxon signed rank test, $p = 0.68$) between the original/pooled (mean = 0.32, median = 0.30, std = 0.49) and the shuffled control datasets (mean = 0.30, median = 0.20, std = 0.30). However, there was a significant difference in their distribution spreads (10,000 bootstraps, 95 % confidence interval). Specifically, 56.8 % of the SEF neurons showed a significant landmark influence relative to their control data (95 % bootstrapped confidence interval). **e** Configuration-dependent Fits. A similar trend as FEF was observed for SEF neurons, with 52.3 % of them being significant for at least one configuration, and different fractions of data being significant (*TLC1-4*: 27.3 % / 25.0 % / 27.3 % / 29.6 %) at the level of individual target-landmark configurations. **f** Recombined data fits. Note that FEF / SEF neurons that were spatially tuned always showed a significantly shifted coding toward both $L_{F(e)}$ and $T_{L(e)}$ for at least one target-landmark configuration, whereas untuned neurons never showed significant shifts along either continuum for any *TLC*. No significant shift was noticed at the population level (Wilcoxon signed rank test, $p = 0.8$). The population fit distributions are shown above and right for all scatter plots.

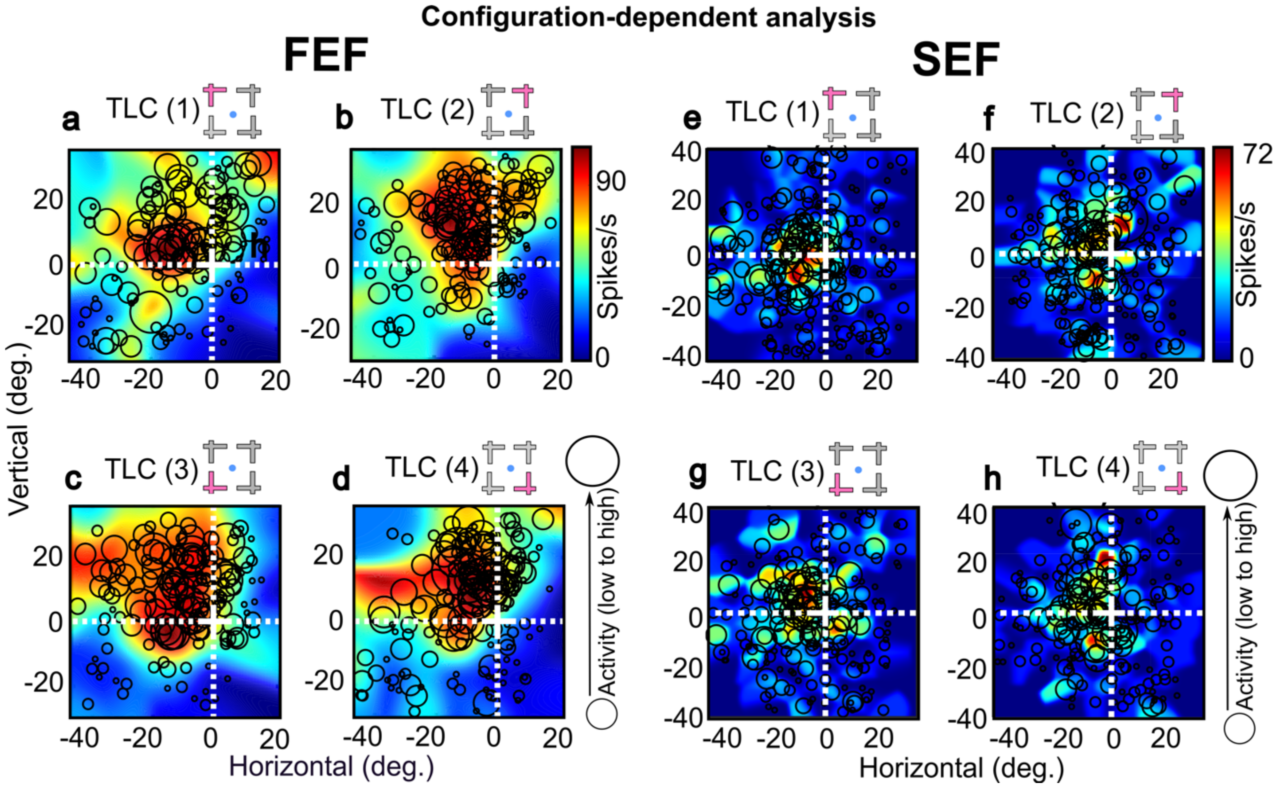

Supplementary Fig. 6: **Configuration-dependent response field analysis for an example FEF / SEF neuron. a-d** Response fields of an example FEF neuron (same neuron as **Fig. 4c-e**) for each target-landmark configuration (TLC1-4), indicated by the magenta cross in the graphic key to the upper-right of each fit relative to the target (solid blue circle). Other conventions are the same as for Figures 4 and 6. In general, this resulted in very similar response fields, differing only in small, variable shifts and distortions (possibly resulting from idiosyncratic sampling biases rather than underlying coordinate systems). **e-h** Same analysis for an example SEF neuron.

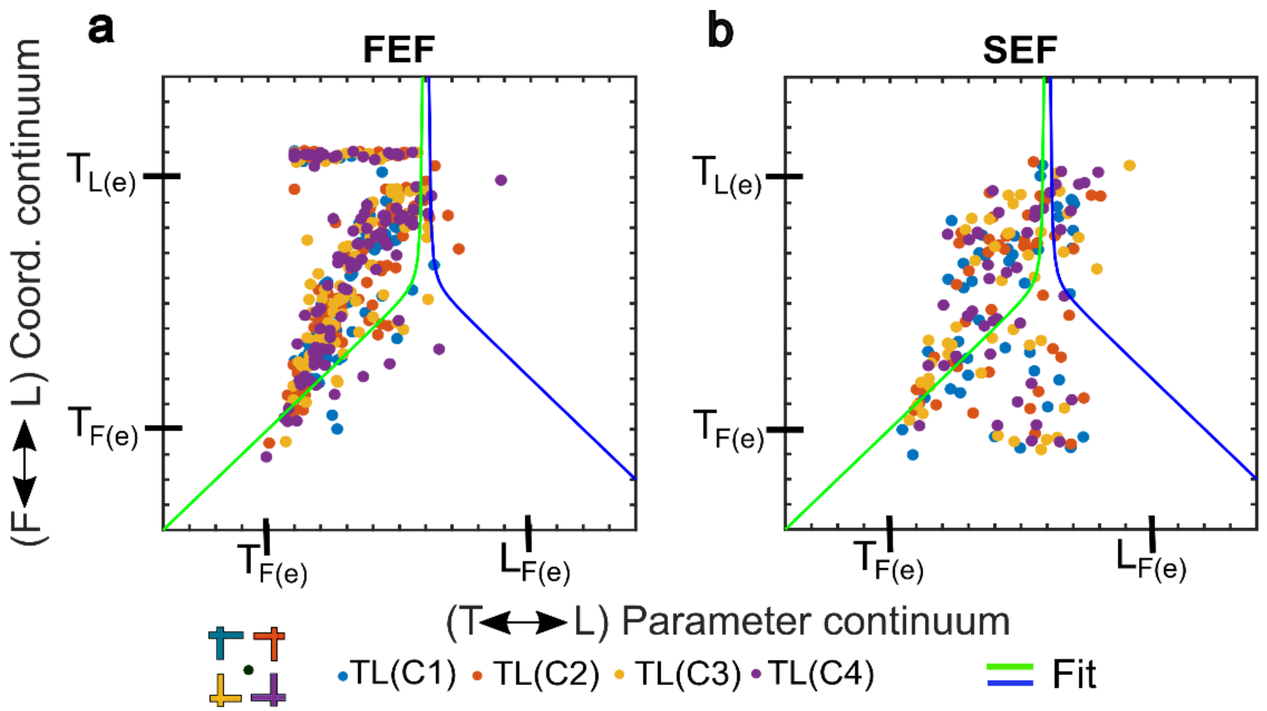

Supplementary Fig. 7: Shuffled-control data for *F-L Coordinate Continuum* scores plotted as a function of *T-L Parameter Continuum* Scores for both FEF **a** and SEF **b**. The asymptotic patterns noticed in **Fig. 8** were not observed in fits to the shuffled control data, suggesting that the results were not an artifact.
